# 103 Analysis of Arterial Blood Pressure Waveform Features in a Porcine Model of Burn and Resuscitation

**DOI:** 10.1093/jbcr/irad045.076

**Published:** 2023-05-15

**Authors:** Ghazal Arabidarrehdor, Yi-Ming Kao, Jin-Oh Hahn, David Burmeister, Babita Parajuli, Bonnie Carney, John Keyloun, Lauren Moffatt, Jeffrey Shupp, Adam Reese, Mary Oliver

**Affiliations:** University of Maryland at College Park, Bethesda, Maryland; University of Maryland, College Park, College Park, Maryland; University of Maryland, College Park, Maryland; Uniformed Services University of the Health Sciences, Bethesda, Maryland; Uniformed Services University of the Health Sciences, Bethesda, Maryland; Firefighters' Burn and Surgical Research Laboratory, Medstar Health Research Institute, Georgetown University Medical Center, Washington, DC; Georgetown University School of Medicine Research, Washington DC, Maryland; Medstar Health, Washington DC, DC; Firefighters' Burn and Surgical Research Laboratory, MedStar Health Research Institute, Georgetown University School of Medicine, Washington, DC; MedStar Georgetown University Hospital-Washington Hospital Center, Washington, DC; Medstar Health, Washington dc, DC; University of Maryland at College Park, Bethesda, Maryland; University of Maryland, College Park, College Park, Maryland; University of Maryland, College Park, Maryland; Uniformed Services University of the Health Sciences, Bethesda, Maryland; Uniformed Services University of the Health Sciences, Bethesda, Maryland; Firefighters' Burn and Surgical Research Laboratory, Medstar Health Research Institute, Georgetown University Medical Center, Washington, DC; Georgetown University School of Medicine Research, Washington DC, Maryland; Medstar Health, Washington DC, DC; Firefighters' Burn and Surgical Research Laboratory, MedStar Health Research Institute, Georgetown University School of Medicine, Washington, DC; MedStar Georgetown University Hospital-Washington Hospital Center, Washington, DC; Medstar Health, Washington dc, DC; University of Maryland at College Park, Bethesda, Maryland; University of Maryland, College Park, College Park, Maryland; University of Maryland, College Park, Maryland; Uniformed Services University of the Health Sciences, Bethesda, Maryland; Uniformed Services University of the Health Sciences, Bethesda, Maryland; Firefighters' Burn and Surgical Research Laboratory, Medstar Health Research Institute, Georgetown University Medical Center, Washington, DC; Georgetown University School of Medicine Research, Washington DC, Maryland; Medstar Health, Washington DC, DC; Firefighters' Burn and Surgical Research Laboratory, MedStar Health Research Institute, Georgetown University School of Medicine, Washington, DC; MedStar Georgetown University Hospital-Washington Hospital Center, Washington, DC; Medstar Health, Washington dc, DC; University of Maryland at College Park, Bethesda, Maryland; University of Maryland, College Park, College Park, Maryland; University of Maryland, College Park, Maryland; Uniformed Services University of the Health Sciences, Bethesda, Maryland; Uniformed Services University of the Health Sciences, Bethesda, Maryland; Firefighters' Burn and Surgical Research Laboratory, Medstar Health Research Institute, Georgetown University Medical Center, Washington, DC; Georgetown University School of Medicine Research, Washington DC, Maryland; Medstar Health, Washington DC, DC; Firefighters' Burn and Surgical Research Laboratory, MedStar Health Research Institute, Georgetown University School of Medicine, Washington, DC; MedStar Georgetown University Hospital-Washington Hospital Center, Washington, DC; Medstar Health, Washington dc, DC; University of Maryland at College Park, Bethesda, Maryland; University of Maryland, College Park, College Park, Maryland; University of Maryland, College Park, Maryland; Uniformed Services University of the Health Sciences, Bethesda, Maryland; Uniformed Services University of the Health Sciences, Bethesda, Maryland; Firefighters' Burn and Surgical Research Laboratory, Medstar Health Research Institute, Georgetown University Medical Center, Washington, DC; Georgetown University School of Medicine Research, Washington DC, Maryland; Medstar Health, Washington DC, DC; Firefighters' Burn and Surgical Research Laboratory, MedStar Health Research Institute, Georgetown University School of Medicine, Washington, DC; MedStar Georgetown University Hospital-Washington Hospital Center, Washington, DC; Medstar Health, Washington dc, DC; University of Maryland at College Park, Bethesda, Maryland; University of Maryland, College Park, College Park, Maryland; University of Maryland, College Park, Maryland; Uniformed Services University of the Health Sciences, Bethesda, Maryland; Uniformed Services University of the Health Sciences, Bethesda, Maryland; Firefighters' Burn and Surgical Research Laboratory, Medstar Health Research Institute, Georgetown University Medical Center, Washington, DC; Georgetown University School of Medicine Research, Washington DC, Maryland; Medstar Health, Washington DC, DC; Firefighters' Burn and Surgical Research Laboratory, MedStar Health Research Institute, Georgetown University School of Medicine, Washington, DC; MedStar Georgetown University Hospital-Washington Hospital Center, Washington, DC; Medstar Health, Washington dc, DC; University of Maryland at College Park, Bethesda, Maryland; University of Maryland, College Park, College Park, Maryland; University of Maryland, College Park, Maryland; Uniformed Services University of the Health Sciences, Bethesda, Maryland; Uniformed Services University of the Health Sciences, Bethesda, Maryland; Firefighters' Burn and Surgical Research Laboratory, Medstar Health Research Institute, Georgetown University Medical Center, Washington, DC; Georgetown University School of Medicine Research, Washington DC, Maryland; Medstar Health, Washington DC, DC; Firefighters' Burn and Surgical Research Laboratory, MedStar Health Research Institute, Georgetown University School of Medicine, Washington, DC; MedStar Georgetown University Hospital-Washington Hospital Center, Washington, DC; Medstar Health, Washington dc, DC; University of Maryland at College Park, Bethesda, Maryland; University of Maryland, College Park, College Park, Maryland; University of Maryland, College Park, Maryland; Uniformed Services University of the Health Sciences, Bethesda, Maryland; Uniformed Services University of the Health Sciences, Bethesda, Maryland; Firefighters' Burn and Surgical Research Laboratory, Medstar Health Research Institute, Georgetown University Medical Center, Washington, DC; Georgetown University School of Medicine Research, Washington DC, Maryland; Medstar Health, Washington DC, DC; Firefighters' Burn and Surgical Research Laboratory, MedStar Health Research Institute, Georgetown University School of Medicine, Washington, DC; MedStar Georgetown University Hospital-Washington Hospital Center, Washington, DC; Medstar Health, Washington dc, DC

## Abstract

**Introduction:**

Functional arterial blood pressure waveform (ABW) features are incompletely understood in the context of burn injury and resuscitation. In this work, we analyzed the variability of pulse pressure (PPV), systolic pressure (SPV), stroke volume (SVV), and five measures of heart rate variability (HRV) in time and frequency domains as potential indicators of burn resuscitation adequacy in a large total body surface area (TBSA) porcine model with varying degrees of intravenous fluids.

**Methods:**

Data were collected from 21 anesthetized and mechanically-ventilated pigs (32±4 kg weight, 40% TBSA), which were instrumented for continuous hemodynamic monitoring for 24 hours. Urinary output (UO), hematocrit (HCT) and cardiac output (CO) were also measured at various time points. The animals were divided into Paradigm 1 (P1): no resuscitation (N=7); Paradigm 2 (P2): fluid titration to UO (N=8); and Paradigm 3 (P3): deliberate over-resuscitation with consistent high fluid rates (N=6). We calculated PPV, SPV, SVV, and five widely used HRV measures: normalized low-frequency power (NLF), high-frequency power (NHF), very-low-frequency power (VLF), low-frequency-high-frequency (LFHF) ratio, and the root-mean-squared of the duration difference in consecutive beats (RR-RMS). All features were averaged on an hourly basis and their potential in tracking reference CO and SV was investigated via linear regression and correlation analysis after calibrating the data to SV and CO on an individual basis.

**Results:**

PPV, SPV, SVV, and RR-RMSE showed statistically distinguishable trends for different paradigms, as depicted in Figure 1, along with averaged trends for lab measurement. PPV, SPV, and SVV tracked the CO the best and were comparable to UO, however, SVV was far superior in tracking SV. A regression analysis between these four features and UO showed a very low level of agreement despite comparable tracking of SV and CO. HRV measures did not distinguish resuscitation paradigms and were inferior in tracking CO and similar in tracking SV to aforesaid features.

**Conclusions:**

PPV, SPV, SVV, and RR-RMSE may potentially complement UO in the hemodynamic assessment of the burn patients and outperform other HRV measures in general. Future work will optimize the extraction of strategic informative features of ABW analysis in the various fluid status states post-burn injury.

**Applicability of Research to Practice:**

Titrating burn resuscitation rates to have protocols including PPV, SPV, SVV, and RR-RMSE alongside other standard metrics may offer precision medicine strategies to fine-tune IV fluid rates.

